# Supplementary material for: A clinically applicable and scalable method to regenerate T-cells from iPSCs for off-the-shelf T-cell immunotherapy
Source: Nat Commun. 2021 Jan 18;12:430. doi: 10.1038/s41467-020-20658-3 (PMC7814014; doi:10.1038/s41467-020-20658-3)
Supplement: Supplementary file 1 — Supplementary Information. [file 41467_2020_20658_MOESM1_ESM.pdf]

**SUPPLEMETARY INFORMATION**

# A clinically applicable and scalable method to regenerate T-cell from iPSCs for off-the-shelf T-cell immunotherapy

**Shoichi Iriguchi et al.**

**Supplementary Figures 1 – 10**
**Supplementary Table 1**

Supplementary Figure 1

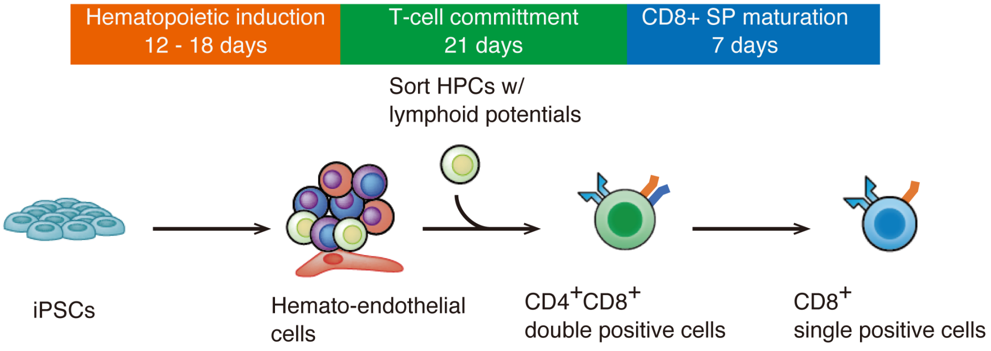


**Supplementary Figure 1. Experimental scheme.** iPSCs were induced to differentiate first into $CD34^+CD43^+$ HPCs for 12–14 days by EB formation. The resulting HPCs were FACS-sorted on DL4-coated culture plates for an additional 21 days to generate $CD4^+CD8^+$ DP-cells. DL4 cells were subsequently maturation cultured in the presence of anti-CD3 antibody for 7 days to induce the generation of $CD4^-CD8\alpha\beta^+$ single positive T-cells (iCD8αβ T-cells). iCD8αβ T-cells were proliferated by CD3 stimulations, and assayed *in vitro* or *in vivo* for their TCR-dependent activities

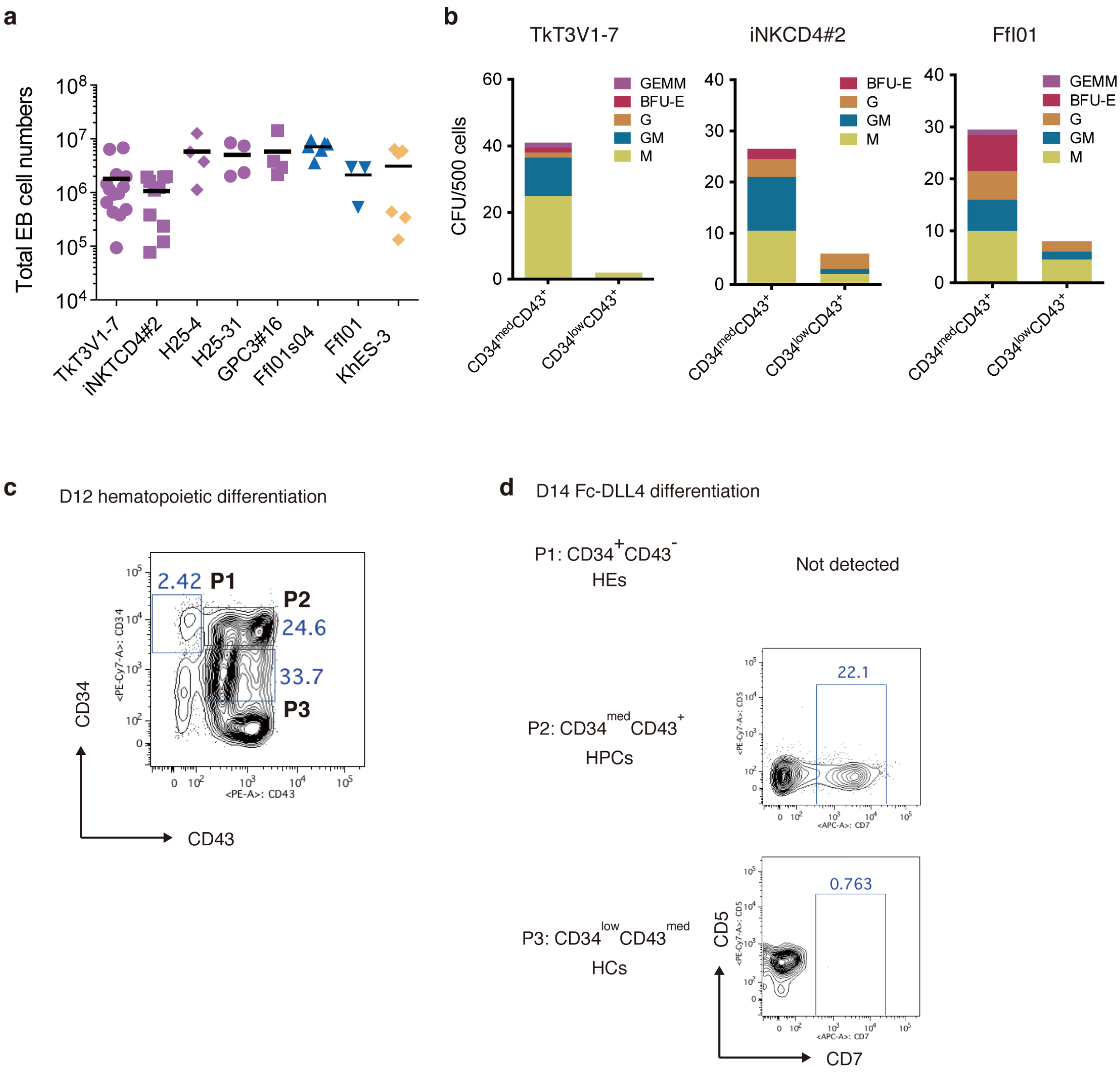
a
Total EB cell numbers
TkT3V1-7
iNKTCD4#2
H25-4
H25-31
GPC3#16
Ff I01s04
Ff I01
KhES-3
b
TkT3V1-7
iNKCD4#2
Ff I01
CFU/500 cells
GEMM
BFU-E
G
GM
M
c
D12 hematopoietic differentiation
2.42 P1
P2
24.6
33.7
P3
CD34
CD43
d
D14 Fc-DLL4 differentiation
P1: CD34+CD43-
HEs
Not detected
P2: CD34medCD43+
HPCs
22.1
P3: CD34lowCD43med
HCs
0.763
CD5
CD7

**Supplementary Figure 2. Characterization of EB-derived iHPCs.** (**a**) Total EB cells derived from multiple human ES/iPSC clones 12–18 days after hematopoietic induction. Dots in purple, blue, and yellow represent iPSC clones derived from T-cells, non-T-cells (proliferated PBMCs), and a human ESC line, respectively. (**b**) Hematopoietic colony-forming potential of the different EB subpopulations. $n = 2$, representative data of independent experiments is shown. (**c** and **d**) T-cell potential of the different EB subpopulations derived from TkT3V1-7 clone measured by the presence of CD7+ cells under Ff culture condition. (**c**) Representative flow diagram showing gating strategy of EB cell fractionations 11 days after differentiation. P1, $CD34^{+}CD43^{-}$; P2, $CD34^{+}CD43^{+}$ HPCs; P3, $CD34^{-}CD43^{+}$. (**d**) Representative flow diagrams of the frequency of CD5 and CD7 14 days after T-cell differentiation. Plots in **c** and **d** are representative of three independent experiments.

Supplementary Figure 3

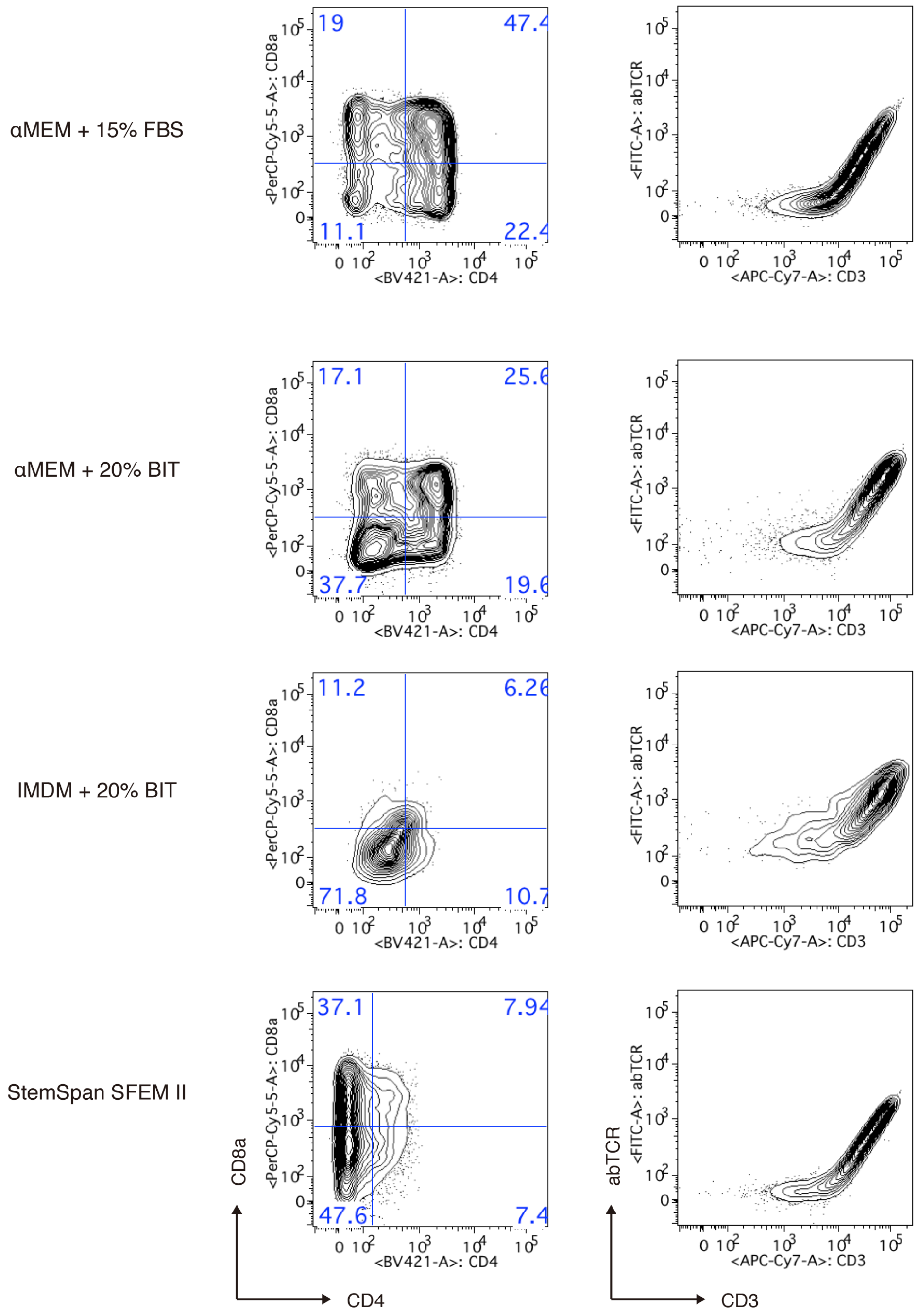

**Supplementary Figure 3. Differentiation of T-cell from iHPCs in serum-free conditions**

Representative flow cytometry plots of T-cell differentiation on DL4 and RN cultures in the presence of SDF1α and SB203580 for 21 days in different serum-free medium. TkT3V1-7-T-iPSC-derived iHPCs were used as input cells.

Supplementary Figure 4

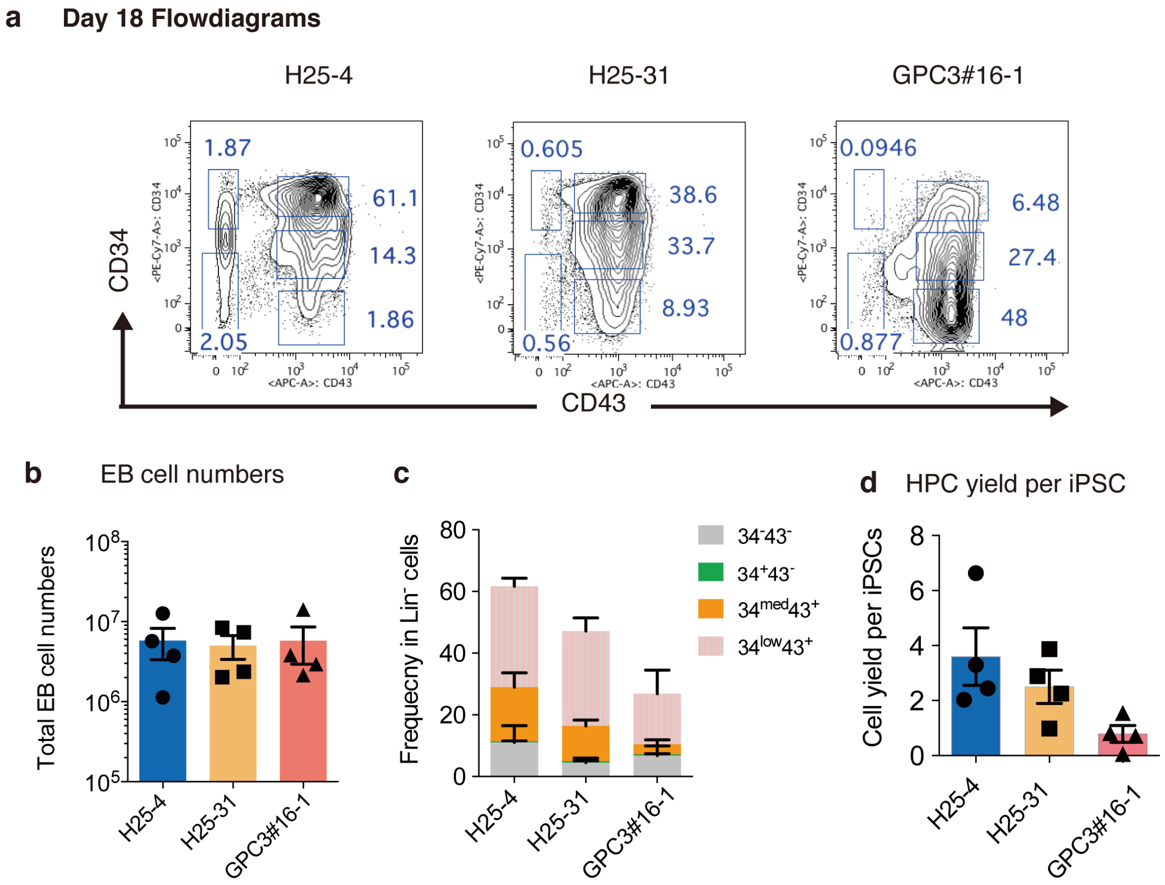


**Supplementary Figure 4. Production of DP-cells from antigen-specific T-iPSC clones by Ff cultures.** (**a**) Representative flow diagrams of the frequency of CD34 and CD43 subpopulations derived from 3 T-iPSC clones 18 days after hematopoietic differentiation. (**b**) Total EB cells derived from T-iPSCs 18 days after differentiation. (*n* = 4 independent experiments) (**c**) Frequency of EB cell subpopulations defined by CD34 and CD43 expression (*n* = 4 independent experiments) (**d**) HPC yields generated from each T-iPSC

clone collected 18 days after differentiation. (*n* = 4 independent experiments). Data represent mean ± SEM of *n* independent experiments.

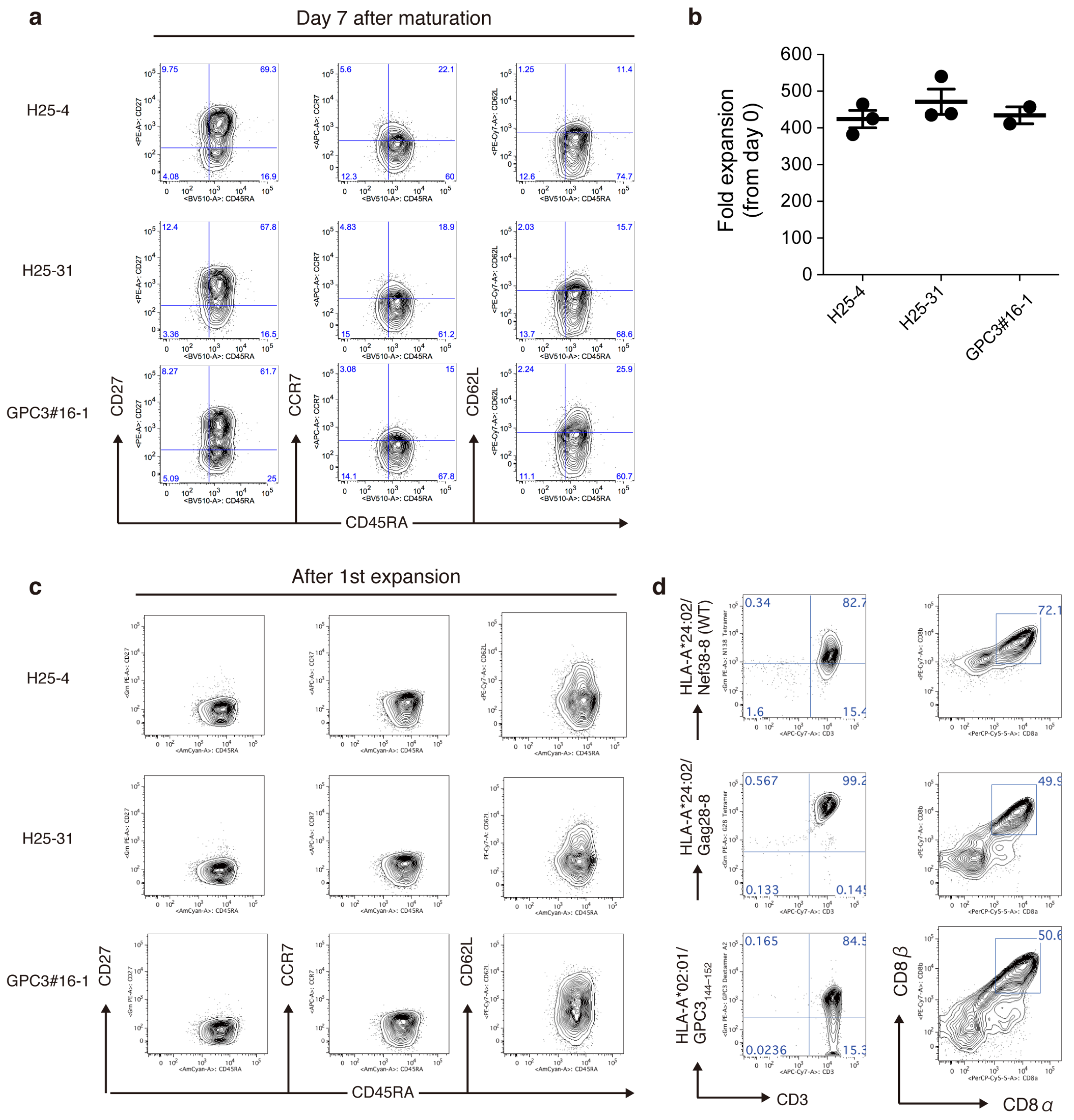


**Supplementary Figure 5. Phenotypes of proliferated iCD8αβ T-cells derived from antigen-specific T-iPSCs.** (**a**) Representative flow cytometry plots obtained 42 days after differentiation (after 7 days in maturation culture as shown in Fig.3**c**) showing

naive/memory T-cell markers, CD45RA, CD27, CCR7, and CD62L of regenerated T-cells (*n* = 3 independent experiments) (**b**) Fold proliferation of tetramer$^+$ CD8αβ$^+$ SP T-cells 14 days after PHA-P/PBMC stimulation in the presence of IL-7 and IL-15. Data represent mean ± SEM of three independent differentiation experiments—except for GPC3#16-1, where two independent differentiation experiments were performed. (**c**) Representative flow cytometry plots obtained 14 days after 1$^{st}$ proliferation showing expression levels of naive/memory T-cell markers, CD45RA, CD27, CCR7, and CD62L. (*n* = 3 independent experiments) (**d**) Flow cytometry plots of proliferated CD8αβ$^+$ SP T-cells showing the expression levels of tetramer and CD3 (Left), and CD8β and CD8α (Right) (*n* = 3 independent experiments).

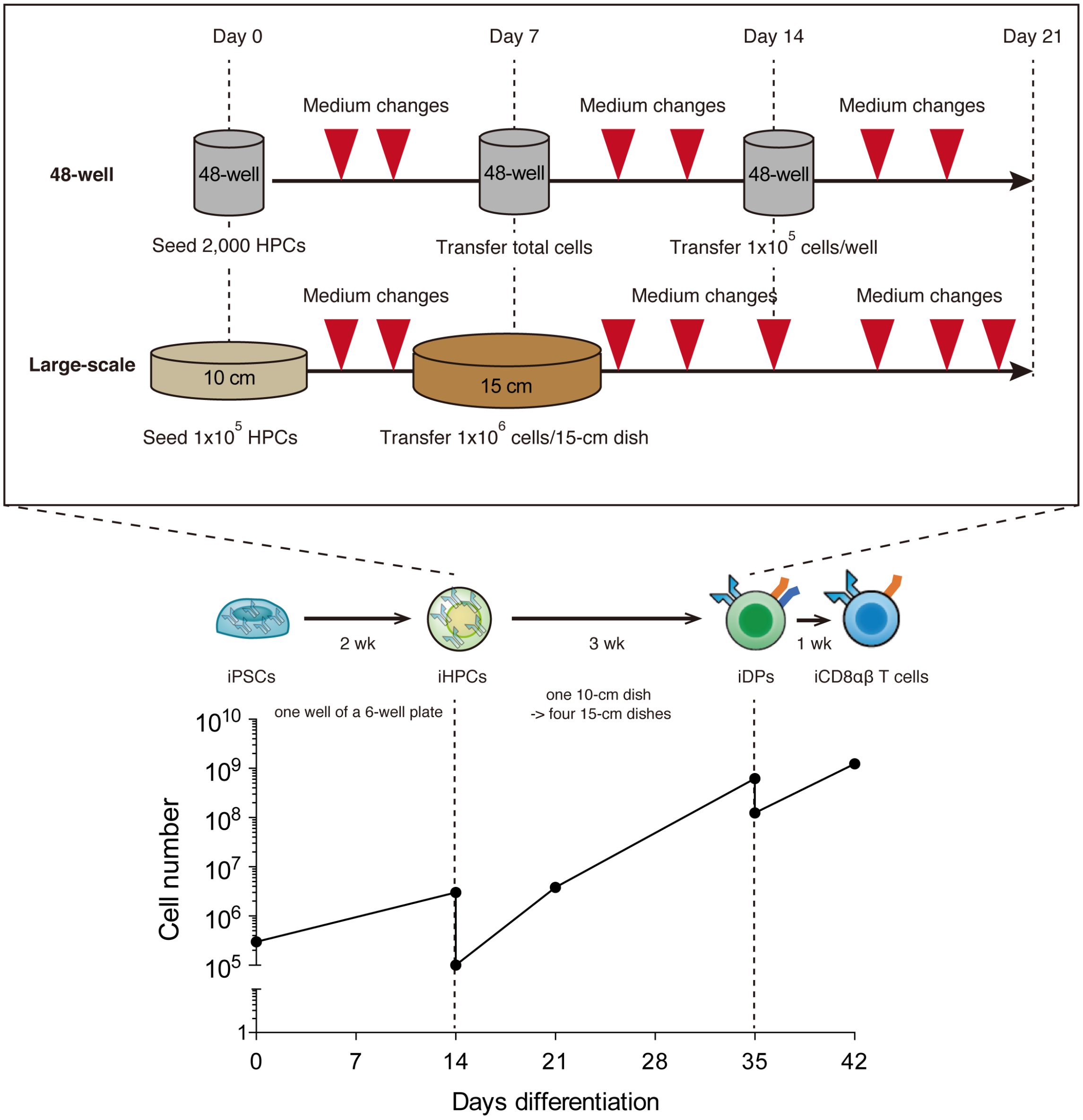
Day 0
Day 7
Day 14
Day 21
Medium changes
Medium changes
Medium changes
48-well
48-well
48-well
48-well
Seed 2,000 HPCs
Transfer total cells
Transfer 1x10^5 cells/well
Medium changes
Medium changes
Medium changes
Large-scale
10 cm
15 cm
Seed 1x10^5 HPCs
Transfer 1x10^6 cells/15-cm dish
iPSCs
2 wk
iHPCs
3 wk
iDPs
1 wk
iCD8αβ T cells
one well of a 6-well plate
one 10-cm dish
-> four 15-cm dishes
Cell number
10^10
10^9
10^8
10^7
10^6
10^5
1
0
7
14
21
28
35
42
Days differentiation

**Supplementary Figure 6** (**Top**) Experimental scheme of Ff T-cell differentiation in small-scale and large-scale cultures. A total of 2000 FACS-sorted $CD34^{+}CD43^{+}$ HPCs were seeded on DL4-coated plate prepared the preceding day and cultured for 7 days with medium being changed every other day. At day 7 and 14, differentiating cells were harvested and seeded on new DL4-coated plates and cultured till day 21. For large-scale cultures, $1\times10^{5}$ FACS-sorted HPCs were seeded on a 10-cm dish pre-coated with DL4 at 4 °C overnight. The medium was completely changed on days 2, 4, 9, 11, 14, 16, 18, 19, and 20. Differentiating cells were harvested and 1 million cells were seeded on a 15-cm dish pre-coated with DL4. Cells were not transferred thereafter. See Methods for more details. (**Bottom**) An example of cell count transition during generation of iCD8αβ T-cells from iPSC staring from one well of a 6-well plate for hematopoietic induction by EB formation. At the end of differentiation culture, we expect to obtain approximately $1.25\times10^{9}$ mature iCD8αβ T-cells in culture vessels.

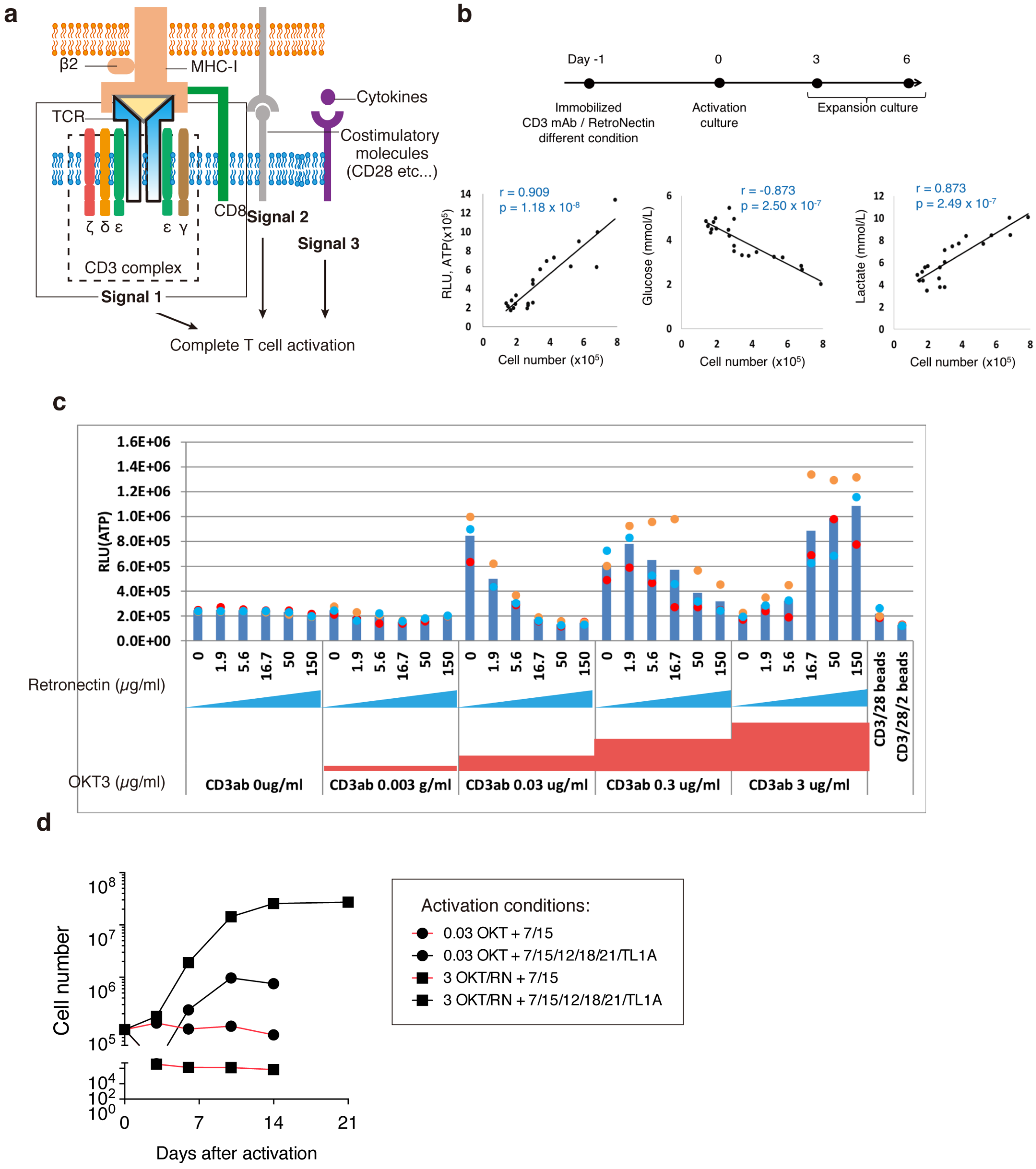
a
β2
MHC-I
TCR
Cytokines
Costimulatory molecules (CD28 etc...)
ζ δ ε
ε γ
CD8
Signal 2
Signal 3
CD3 complex
Signal 1
Complete T cell activation
b
Day -1
0
3
6
Immobilized CD3 mAb / RetroNectin different condition
Activation culture
Expansion culture
r = 0.909
p = 1.18 x 10-8
RLU, ATP(x10⁵)
Cell number (x10⁵)
r = -0.873
p = 2.50 x 10-7
Glucose (mmol/L)
r = 0.873
p = 2.49 x 10-7
Lactate (mmol/L)
c
RLU(ATP)
Retronectin (μg/ml)
OKT3 (μg/ml)
CD3ab 0ug/ml
CD3ab 0.003 g/ml
CD3ab 0.03 ug/ml
CD3ab 0.3 ug/ml
CD3ab 3 ug/ml
CD3/28 beads
CD3/28/2 beads
d
Cell number
Days after activation
Activation conditions:
0.03 OKT + 7/15
0.03 OKT + 7/15/12/18/21/TL1A
3 OKT/RN + 7/15
3 OKT/RN + 7/15/12/18/21/TL1A

**Supplementary Figure 7. Optimization of iCD8αβ T-cell proliferation culture conditions.** (**a**) A schematic diagram illustrating the components of TCR/CD3 signal complex (Signal 1), costimulatory molecules such as CD28 (Signal 2), and cytokine signaling (Signal 3) involved in the complete T-cell activation. (**b**) Assessment of surrogate markers for T-cell proliferation for identification of the optimum concentrations of CD3, mAb and retronectin (RN). ATP, glucose, and lactate concentrations were compared to cell count 3 days after stimulation (at the end of activation culture). ATP concentration was chosen as a surrogate marker for this purpose. ($n$ = 2 independent experiments) (two-sided Student's t-tests) (**c**) ATP concentrations 12 days after stimulation with varying OKT3 and RN concentrations. Data represent mean ± SEM ($n$ = 1 independent experiment) (**d**) Growth curve of iCD8αβ cells after stimulation with different OKT3 and RN concentrations in the presence or absence of the separately identified combination of cytokines (IL-12, -18, -21, and TL-1A). ($n$ = 2 independent experiments)

Supplementary Figure 8

**a**

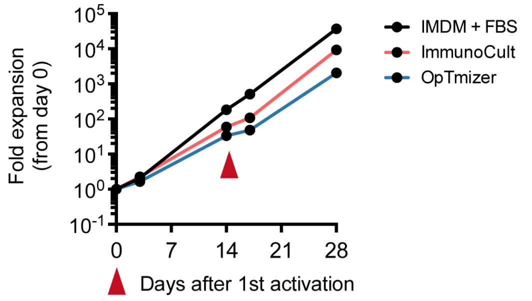


| Days after 1st Activation | Medium | | |
|---|---|---|---|
| | IMDM + FBS | ImmunoCult | OpTimizer |
| 0 | 1 | 1 | 1 |
| 3 | 2.1 | 2.3 | 1.7 |
| 14 | 183.6 | 59.2 | 33.1 |
| 17 | 510.4 | 107.5 | 48.3 |
| 28 | 36995.4 | 9262.7 | 2060.6 |

**b**

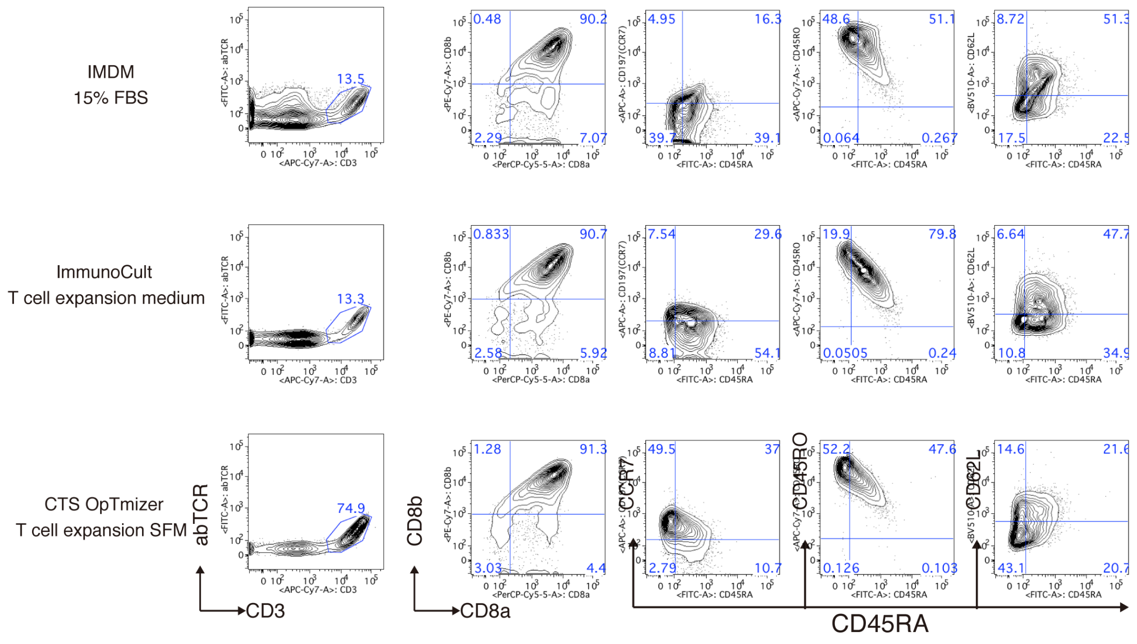


**Supplementary Figure 8.** ***Proliferation of*** **WT1-TCR-transduced HLA-homo iCD8αβ T-cells in serum-free conditions.** (**a**) Fold proliferation of WT1-TCR iCD8αβ T-cells in an optimized protocol as shown Figure 5 for two sequential rounds of stimulations in the indicated medium. (**b**) Flow cytometry plots of proliferated WT1-iPSC CD8 SP T-cells in

the indicated medium representing the expression levels of CD3, αβTCR, CD8α, CD8β and naive/memory T-cell markers.

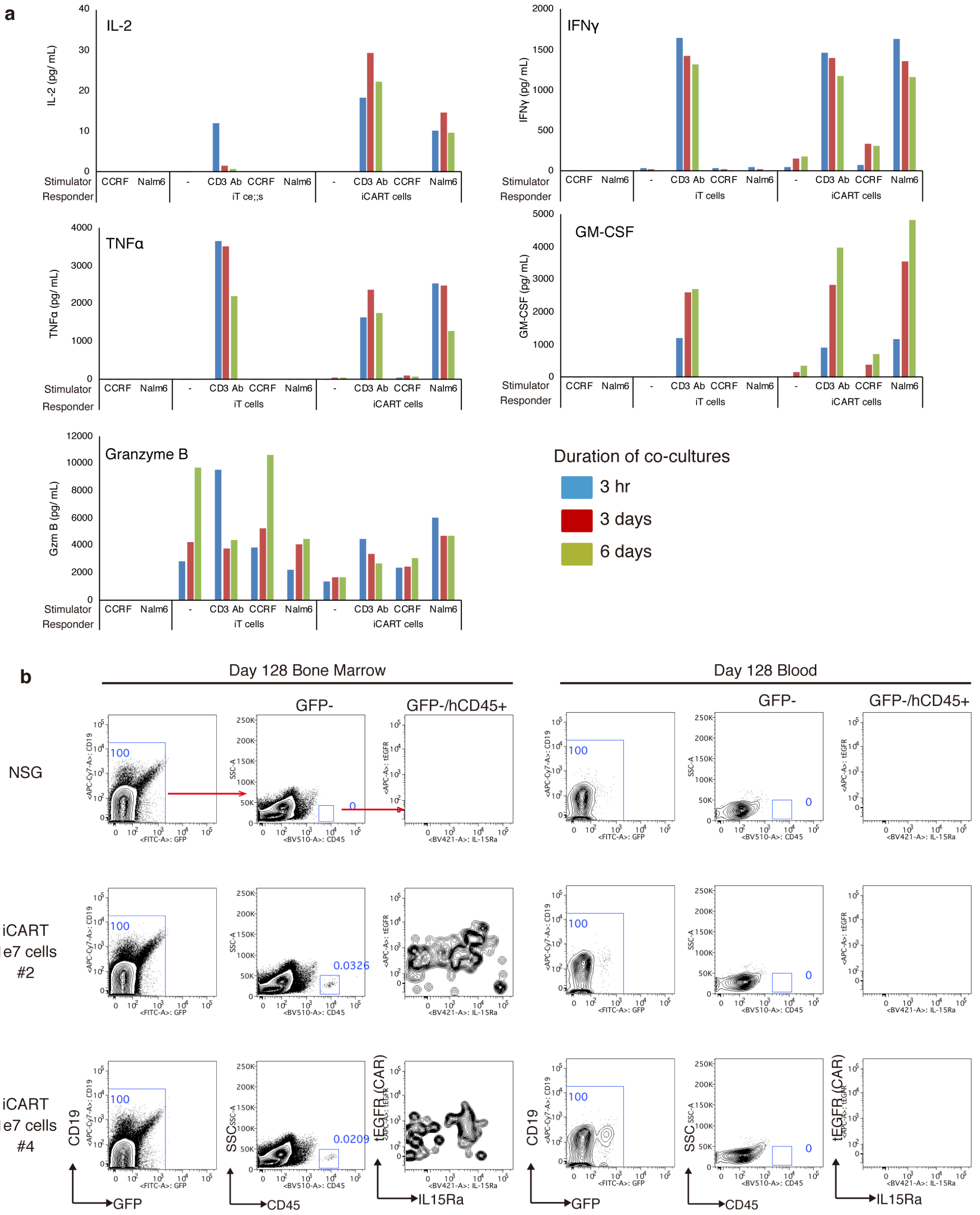
a
IL-2
IL-2 (pg/ mL)
IFNγ
IFNγ (pg/ mL)
TNFα
TNFα (pg/ mL)
GM-CSF
GM-CSF (pg/ mL)
Granzyme B
Gzm B (pg/ mL)
Stimulator
Responder
CCRF
Nalm6
-
CD3 Ab
iT ce;;s
iT cells
iCART cells
Duration of co-cultures
3 hr
3 days
6 days
b
Day 128 Bone Marrow
Day 128 Blood
GFP-
GFP-/hCD45+
NSG
iCART 1e7 cells #2
iCART 1e7 cells #4
CD19
GFP
SSC
CD45
tEGFR (CAR)
IL15Ra
100
0
0.0326
0.0209

**Supplementary Figure 9. *In vitro* and *in vivo* functions of iCART cell.** (**a**) CD19-dependent cytokine production of iT cells and iCART cells. Concentrations of IL-2, IFNγ, TNF, GM-CSF, and granzyme B in the culture supernatants collected at the indicated days from the cocultures. (**b**) NALM-6-bearing mice were treated with $1x10^7$ iCART cells. At 128 after treatment, the relapse-free mice were euthanized and bone marrow cells and blood cells were collected. Presence of iT cell or iCART cells ($GFP^-CD19^-humanCD45^+$) and NALM-6 ($GFP^+CD19^+$) cells were analyzed by flow cytometry. Expression levels of CAR and IL-15Rα in the human $CD45^+$ cells are also shown (Right).

Supplementary Figure 10

a

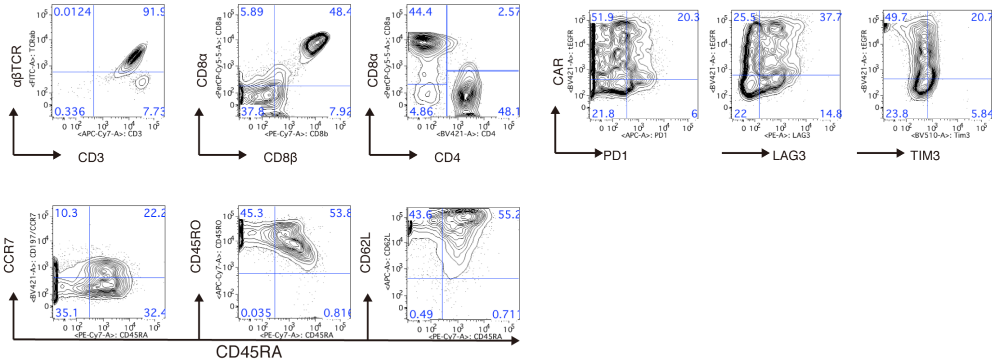


b

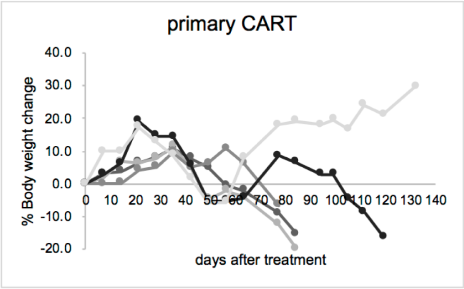


**Supplementary Figure 10. Generation and assessment of 19BBz from primary T cells.**

(**a**) Representative flow cytometry plots of 19BBz-transduced primary T cells (pCART cells) showing expression levels of CD3, αβTCR, CD8α, CD8β (Top left), naive/memory T-cell markers (Bottom left) and exhaustion markers (Right). (**b**) Percentage of body weight changes of mice treated with primary CART cells over time. Each line represents one recipient. Mice exhibited more than 20% weight loss were euthanized in this study.

**Supplementary Table 1.** The list of TaqMan assay IDs used in this study.

| GENE Symbol | Assay ID |
|---|---|
| **TCF3** | Hs00413032_m1 |
| **GATA3** | Hs00231122_m1 |
| **BCL11B** | Hs01102259_m1 |
| **TCF7** | Hs00175273_m1 |
| **NOTCH1** | Hs01062014_m1 |
| **HES1** | Hs00172878_m1 |
| **DELTEX** | Hs01092201_m1 |
| **SPI1** | Hs02786711_m1 |
| **ACTB** | Hs01060665_g1 |
