# Supplementary material for: A clinically applicable and scalable method to regenerate T-cells from iPSCs for off-the-shelf T-cell immunotherapy
Source: Nat Commun. 2021 Jan 18;12:430. doi: 10.1038/s41467-020-20658-3 (PMC7814014; doi:10.1038/s41467-020-20658-3)
Supplement: Supplementary file 3 — Reporting Summary [file 41467_2020_20658_MOESM3_ESM.pdf]

nature research

Corresponding author(s): Shin Kaneko

Last updated by author(s): Nov 12, 2020

Reporting Summary

Nature Research wishes to improve the reproducibility of the work that we publish. This form provides structure for consistency and transparency in reporting. For further information on Nature Research policies, see Authors & Referees and the Editorial Policy Checklist .

Statistics

For all statistical analyses, confirm that the following items are present in the figure legend, table legend, main text, or Methods section.

n/a	Confirmed	
☐	☒	The exact sample size (*n*) for each experimental group/condition, given as a discrete number and unit of measurement
☐	☒	A statement on whether measurements were taken from distinct samples or whether the same sample was measured repeatedly
☐	☒	The statistical test(s) used AND whether they are one- or two-sided<br>*Only common tests should be described solely by name; describe more complex techniques in the Methods section.*
☐	☒	A description of all covariates tested
☐	☒	A description of any assumptions or corrections, such as tests of normality and adjustment for multiple comparisons
☐	☒	A full description of the statistical parameters including central tendency (e.g. means) or other basic estimates (e.g. regression coefficient) AND variation (e.g. standard deviation) or associated estimates of uncertainty (e.g. confidence intervals)
☐	☒	For null hypothesis testing, the test statistic (e.g. *F*, *t*, *r*) with confidence intervals, effect sizes, degrees of freedom and *P* value noted<br>*Give P values as exact values whenever suitable.*
☒	☐	For Bayesian analysis, information on the choice of priors and Markov chain Monte Carlo settings
☒	☐	For hierarchical and complex designs, identification of the appropriate level for tests and full reporting of outcomes
☒	☐	Estimates of effect sizes (e.g. Cohen's *d*, Pearson's *r*), indicating how they were calculated

Our web collection on statistics for biologists contains articles on many of the points above.

Software and code

Policy information about availability of computer code

Data collection	FACS Diva 8.0.1 (Flow cytometry), QuantStudio Real-time PCR Software v1.2 (real-time PCR), the Living image Software V4.2 (in vivo imaging), EnVision Manager 1.13 (in vitro cytotoxicity assays), FCAP array v. 3.0.19 (CBA)
Data analysis	Flowjo 9.9.6, Prism 6, Microsoft Excel for Mac 16.16.27

For manuscripts utilizing custom algorithms or software that are central to the research but not yet described in published literature, software must be made available to editors/reviewers. We strongly encourage code deposition in a community repository (e.g. GitHub). See the Nature Research guidelines for submitting code & software for further information.

Data

Policy information about availability of data

All manuscripts must include a data availability statement. This statement should provide the following information, where applicable:

- Accession codes, unique identifiers, or web links for publicly available datasets
- A list of figures that have associated raw data
- A description of any restrictions on data availability

The authors declare that all data that support the findings of this study are available in this article and are provided as a Source File Data file or from the corresponding author upon reasonable request.

Field-specific reporting

Please select the one below that is the best fit for your research. If you are not sure, read the appropriate sections before making your selection.

Life sciences study design

All studies must disclose on these points even when the disclosure is negative.

Sample size	No statistical methods were used to predetermine the experimental sample size. The sample size was determined based on prior literature in this field (e.g., Kennedy et al., Cell Reports, 2013).
Data exclusions	No data were excluded.
Replication	For all figures, two or three independent experiments were performed and all attempts at replicating observation were successful. Similar results were obtained across two laboratories. The replication number is indicated in the legend of corresponding figures.
Randomization	All samples were number coded until the measurement was completed. For in vivo experiments, mice were randomly assigned to each group.
Blinding	Blinding was not performed. Fully blinded experiments were not possible due to personnel availability to accommodate such experiments.

Reporting for specific materials, systems and methods

We require information from authors about some types of materials, experimental systems and methods used in many studies. Here, indicate whether each material, system or method listed is relevant to your study. If you are not sure if a list item applies to your research, read the appropriate section before selecting a response.

Materials & experimental systems

n/a	Involved in the study	
☐	☒	Antibodies
☐	☒	Eukaryotic cell lines
☒	☐	Palaeontology
☐	☒	Animals and other organisms
☐	☒	Human research participants
☒	☐	Clinical data

Methods

n/a	Involved in the study	
☒	☐	ChIP-seq
☐	☒	Flow cytometry
☒	☐	MRI-based neuroimaging

Antibodies

Antibodies used

For tetramer co-staining, PE- or APC-conjugated HLA-A*02:01–GPC3157–165, HLA-A*02:01–HIV26–35, HLA-A*24:02-WT-1235-243 (Immudex), and HLA-A*24:02 Nef, HLA-A*24:02-Gag tetramers (MBL International) were used.
APC/Cy7 Anti-human CD3 UCHT1 Biolegend 300426 (1:100)
BV421 Anti-human CD4 OKT4 Biolegend 317434 (1:100)
PE/Cy7 Anti-human CD5 UCHT2 eBioscience 25-0059-42 (1:100)
APC Anti-human CD7 CD7-6B7 Biolegend 343108 (1:100)
PerCP/Cy5.5 Anti-human CD8a SK1 Biolegend 344710 (1:100)
PE Anti-human CD8b 2ST8.5H7 BeckmanCoulter IM2217U (1:100)
APC/eF780 Anti-human CD14 61D3 eBioscience 470149-42 (1:100)
PE Anti-human CD27 O323 Biolegend 302808 (1:100)
PE/Cy7 Anti-human CD34 4H11 Abcam ab155358 (1:200)
APC Anti-human CD43 1G10 BD Pharmingen 560198 (3:100)
Brilliant Violet 510 Anti-human CD45 HI30 Biolegend 304036 (1:100)
FITC Anti-human CD45RA HI100 Biolegend 304106 (1:100)
APC/Cy7 Anti-human CD45RO UCHL1 Biolegend 304228 (1:100)
BV510 Anti-human CD62L DREG-56 Biolegend 304844 (1:100)
AF647 Anti-human CCR7 Go43H7 Biolegend 353218 (1:100)
FITC Anti-human CD235a GA-R2 (HIR2) BD Pharmingen 559943 (1:100)
FITC Anti-human IFN-gamma B27 Biolegend 506504 (1:100)
APC Anti-human IL-2 MQ1-17H12 Biolegend 500310 (1:100)
BV421 Anti-human TNFalpha MAb11 Biolegend 502932 (1:100)
FITC Anti-human abTCR WT31 eBioscience 11-9955-42 (1:100)

Validation

Antibodies were validated using positive and negative cells using human PBMCs or isotype controls. (CD45RA, CD45RO, CCR7, CD62L; Figures 4, 5 and 6, Supplementary Figures 5, 8 and 10.)
Validation reports were also provided by the antibody manufacturers (BioLegend and BD biosciences). Compensation controls were used for every experiment. The BD Fortessa and Aria Fusion were calibrated daily using CS&T beads (BD Biosciences).
https://www.biolegend.com/ja-jp/products/apc-cyanine7-anti-human-cd3-antibody-3929
https://www.biolegend.com/ja-jp/products/brilliant-violet-421-anti-human-cd4-antibody-7775

https://www.thermofisher.com/antibody/product/CD5-Antibody-clone-UCHT2-Monoclonal/25-0059-42
https://www.biolegend.com/ja-jp/products/apc-anti-human-cd7-antibody-6088
https://www.biolegend.com/ja-jp/products/percp-cyanine5-5-anti-human-cd8-antibody-6389
https://www.beckman.jp/reagents/coulter-flow-cytometry/antibodies-and-kits/single-color-antibodies/cd8beta/im2217u
thermofisher.com/antibody/product/CD14-Antibody-clone-61D3-Monoclonal/47-0149-42
https://www.biolegend.com/ja-jp/products/pe-anti-human-cd27-antibody-811
https://www.abcam.co.jp/cd34-antibody-4h11-prediluted-pecy7-ab155358.html
https://www.bdbiosciences.com/jp/applications/research/b-cell-research/surface-markers/human/apc-mouse-anti-human-cd43-1g10/p/560198
https://www.biolegend.com/ja-jp/products/brilliant-violet-510-anti-human-cd45-antibody-8006
https://www.biolegend.com/ja-jp/products/fitc-anti-human-cd45ra-antibody-686
https://www.biolegend.com/ja-jp/products/apc-cyanine7-anti-human-cd45ro-antibody-7372
https://www.biolegend.com/ja-jp/products/brilliant-violet-510-anti-human-cd62l-antibody-13426
https://www.biolegend.com/ja-jp/products/alexa-fluor-647-anti-human-cd197-ccr7-antibody-7538
https://www.bdbiosciences.com/jp/reagents/research/antibodies-buffers/immunology-reagents/anti-human-antibodies/cell-surface-antigens/fitc-mouse-anti-human-cd235a-ga-r2-hir2/p/559943
https://www.biolegend.com/ja-jp/products/fitc-anti-human-ifn-gamma-antibody-1534
https://www.biolegend.com/ja-jp/products/apc-anti-human-il-2-antibody-1348
https://www.biolegend.com/ja-jp/products/brilliant-violet-421-anti-human-tnf-alpha-antibody-7215
https://www.thermofisher.com/antibody/product/TCR-alpha-beta-Antibody-clone-WT31-Monoclonal/11-9955-42

Eukaryotic cell lines

Policy information about cell lines

Cell line source(s)	B-LCL (RIKEN Cell bank), SK-Hep-1 (ATCC), NCI-H226 (ATCC), NALM6 (RIKEN Cell bank), CCRF-CRM (ATCC)
Authentication	None of the cell lines used have been authenticated.
Mycoplasma contamination	All cell lines were confirmed negative to mycoplasma contamination
Commonly misidentified lines (See ICLAC register)	None of the cell lines used in this manuscript are listed in the ICLAC Database of Cross-contaminated or Misidentified Cell Lines.

Animals and other organisms

Policy information about studies involving animals; ARRIVE guidelines recommended for reporting animal research

Laboratory animals	NOD.Cg-PrkdcscidIl2rgtm1Sug/ShiJic (NOG) mice (Female, 6w) were obtained from CIEA (Kanagawa, Japan). Mice were exposed to 12:12 h light-dark cycle with free access to water and food. The ambient temperature was restricted to 20-26 degrees C and the room humidity ranged from 40-70%
Wild animals	The study did not involve any wild animals
Field-collected samples	The study did not involve any samples collected from the field.
Ethics oversight	All animal experiments were conducted under a protocol approved by the institutional Animal Research Committee at Takeda Pharmaceutical Company Ltd..

Note that full information on the approval of the study protocol must also be provided in the manuscript.

Human research participants

Policy information about studies involving human research participants

Population characteristics	Healthy donor PBMCs and T cells were used.
Recruitment	PBMCs were purchased from Precision for Medicine. T cells were isolated from the PBMCs.
Ethics oversight	The use of the purchased PBMCs was approved by ethics committees at Takeda Pharmaceutical Company Ltd.

Note that full information on the approval of the study protocol must also be provided in the manuscript.

Flow Cytometry

Plots

Confirm that:

- [x] The axis labels state the marker and fluorochrome used (e.g. CD4-FITC).
- [x] The axis scales are clearly visible. Include numbers along axes only for bottom left plot of group (a 'group' is an analysis of identical markers).
- [x] All plots are contour plots with outliers or pseudocolor plots.
- [x] A numerical value for number of cells or percentage (with statistics) is provided.

Methodology

Sample preparation	All flow cytometry staining were performed in MACS buffer (0.5% BSA and 2 mM EDTA in PBS) for 30 min on ice. For tetramer co-staining, PE- or APC-conjugated HLA-A*02:01–GPC3157–165, HLA-A*02:01–HIV26–35, HLA-A*24:02-WT-1235-243 (Immudex), and HLA-A*24:02 Nef, HLA-A*24:02-Gag tetramers (MBL International) were added to cells at a 1:20 final dilution and stained for 30 min on ice. After washing, cells were stained with additional antibodies for 20 min on ice. PI was added to all samples before analysis. LSRII Fortessa and FACS AriaII or Aria Fusion instrument (BD Biosciences, San Jose, CA) were used for flow cytometry analysis and cell sorting, respectively
Instrument	FACS Aria II and FACS Aria Fusion
Software	FACS Diva8.0 and Flowjo 9.6.6
Cell population abundance	A portion of FACS or MACS-sorted cells was re-analyzed by flowcytometry (FACS Aria Fusion) and confirmed to > 97% purity for FACS-sorting and >90% for MACS-sorting, respectively.
Gating strategy	Gating was performed as follows: FSC/SSC -> Singlets -> Live cells (PI negative) -> gating of interest. The appropriate negative control was used for generating gates of interest. For instance, T cell memory phenotypes were gated based on staining prepared by human PBMCs. For TCR positivity, non TCR transduced iPSC lines were differentiated and analyzed side by side to draw gating.

- [x] Tick this box to confirm that a figure exemplifying the gating strategy is provided in the Supplementary Information.
